# Derivation and Validation of a Mortality Risk Score for Severe Hand, Foot and Mouth Disease in China

**DOI:** 10.1038/s41598-017-02658-4

**Published:** 2017-06-13

**Authors:** Jun Qiu, Xiulan Lu, Xiao Liu, Ping Zang, Wenjiao Zhao, Pingping Liu, Zhenghui Xiao

**Affiliations:** Emergency Center, Hunan Children’s Hospital, Changsha, China

## Abstract

Outbreaks of hand, foot and mouth disease (HFMD) have increased recently, as has the case fatality rate in severe cases. No scoring system currently exists to predict mortality risk for severe HFMD in previous study. We retrospectively collected laboratory parameters for 546 patients with severe HFMD (a derivation and a validation cohort) at Hunan Children’s Hospitals between January 2012 and December 2014. We developed a mortality risk score comprising four laboratory parameters: blood glucose (GLU), white blood cells (WBC), lactate (LAC), and N-terminal-probrain natriuretic peptide (NT-proBNP). Using an “optimal” cutoff score of 4, the sensitivity, specificity, positive predictive value and negative predictive value was 88.00%, 96.14%, 62.86% and 99.08%, respectively, in the derivation cohort. Among severe HFMD patients with low- and high-risk scores in the validation cohort, case fatality rates were 1.49% and 74.07%, respectively. According to the “optimal” cut-off in the derivation cohort, the sensitivity, specificity, positive predictive value, and negative predictive value were 80.95%, 93.83%, 62.96% and 97.44%, respectively, in the derivation cohort. The mortality risk score demonstrated good discrimination (AUC > 0.9) and calibration (P > 0.05) in both cohorts. The mortality risk score, comprising WBC, GLU, LAC and NT-proBNP, has been demonstrated good discrimination and calibration in the both cohorts.

## Introduction

Hand, foot and mouth disease (HFMD), a common infectious disease among infants and children, is mainly caused by enterovirus 71(EV71) and coxsackievirus A16 (CA16). It is typically characterized by fever with mouth ulcers and eruption of vesiculo-papular rash over the hands, soles, and/or buttocks. Most cases are not life-threatening; however, a small proportion of HFMD patients have a poor prognosis, especially those infected with EV71, owing to complications such as aseptic meningitis, encephalitis, pulmonary oedema, and cardiopulmonary haemorrage^1^. In 2008, large HFMD outbreaks and epidemics occurred in Anhui Province of China, resulting in approximately 490,000 infection cases and 126 deaths among infants and young children^2^. Since then, the Chinese government has listed HFMD as a nationally notifiable class C infectious disease and established a national enhanced surveillance system for HFMD. In recent years, HFMD outbreaks have been increasingly reported in eastern and southeastern Asian countries and regions^3–5^. In Hunan Province of China, the incidence rate of HFMD was increased to 318.05/100000 population in 2014 from 287.32/100000 population in 2012^6, 7^. Thus, HFMD is considered a major public health problem in China, and seriously threat to the health of Chinese children.

Together with the recent increase of HFMD outbreaks, increasingly severe cases of HFMD have also appeared^8, 9^. Furthermore, reports on HFMD epidemics support that the case fatality rate among severe cases is increasing^10^. Such fatal cases are usually noted in young infants, and are characterized by serious complications, such as central nervous system manifestations, pulmonary oedema, pulmonary haemorrhage, circulatory failure, and rapid shock^11^. These conditions progress very quickly, and rapid disease progression can lead to death within 24 to 48 hours, making early detection difficult. In a recently published meta-analysis, fever lasting for more than 3 days, body temperature over 37.5 °C, lethargy, hyperglycaemia, vomiting, increased neutrophil count, EV71 infection, and young age are risk factors for severe HFMD^9^. Similarly, many studies have demonstrated that young age, peak temperature of 38.5 °C or more, vomiting, circulatory disturbance, respiratory rhythm dysfunction, hyperglycaemia, and leukocytosis are independent risk factors for severe HFMD complicated by cardiopulmonary collapse^12^ Previous studies have demonstrated that patients with EV-71 infection or symptoms of convulsion, dyspnoea, cyanosis, coolness of extremities, and vomiting had an increased risk of death owing to severe HFMD^13^. Some common models for encephalitis and the consequent occurrence of pulmonary edema or cardiopulmonary collapse have been developed in recent previous studies^12, 14^. However, there are several drawbacks of these models. First, these models included several subjective clinical manifestations and objective physiological parameters. “Gold standard” clinical manifestations, such as lethargy or circulatory disturbance, are not identified in a timely and effectively manner, for junior doctors. Further, these models cannot accurately calculate the probability of death from clinical manifestations and objective physiological parameters. Finally, prediction models in paediatric intensive care units, such as PRISM (Pediatric Risk of Mortality score) or PCIS (Pediatric Critical Illness Score), cannot adequately evaluate the severity or mortality risk of HFMD^15, 16^. Therefore, determining the mortality risk score using laboratory parameters for HFMD would be an important way to provide effective and timely medical interventions, and reduce the mortality rate of severe HFMD.

The purpose of this study was to develop a mortality risk score based on laboratory parameters collected at the time of admission for children with severe HFMD.

## Results

### Patient characteristics

In total, 546 patients with severe HFMD were recruited in this study, with a sex ratio of 1.76:1 (348/198). The median age of the study cohort was 21 months (IQR:15~30 months). There were 456 (83.52%) patients aged less than 3 years and 90 (16.48%) aged 3 years or older. A total of 414 (75.82%) HFMD cases were caused by EV71, and 132 (24.18%) were caused by CA16 or other enterovirus. and the mortality rate was 8.42% (46/546). There were 126 (23.08%) cases with stage II, 107 (19.60%) cases with stage III, and 31 (5.68) cases with stage IV. The length of hospital stay was 8.69 ± 5.15 days and the length of ICU stay was 6.62 ± 4.34 days. Patients were divided into a derivation cohort (362 cases) and a validation cohort (184 cases). The baseline clinical characteristics of patients in the derivation cohort and validation cohort are shown in the Table 1. There were no significant differences between the two cohorts (P > 0.05) expect with respect to length of stay in the hospital.

**Table 1:** Baseline characteristics of severe HFMD patients in the derivation and validation cohort.

Variables	Derivation cohort(n = 362)	Validation cohort(n = 184)	t/*χ* ^2^	*P*
Age group				
≤3ys	306(84.53)	150(81.52)	0.80	0.37
>3ys	56(15.47)	34(18.48)		
Gender				
Male	230(63.54)	118(64.13)	0.02	0.89
Female	132(36.46)	66(35.87)		
Enteroviruses				
EV-71	282(77.90)	132(71.74)	2.53	0.11
Non-EV71	80(22.10)	52(28.26)		
Stage				
II	41(51.25)	85(46.20)	0.58	0.75
III	30(37.50)	77(41.85)		
IV	9(11.25)	22(11.96)		
Complications				
Brainstem encephalitis	254(70.17)	137(74.46)	1.11	0.29
Pulmonary edema	43(11.88)	30(16.30)	2.06	0.15
Pulmonary hemorrhage	33(9.12)	24(13.04)	2.01	0.16
Circulatory failure	31(8.56)	24(13.04)	2.70	0.10
Length of stay	7.62 ± 4.07	10.79 ± 6.30	−6.20	<0.001
Length of ICU stay	6.71 ± 3.61	6.43 ± 5.52	0.72	>0.05
Outcome				
Survivor	337(93.09)	163(88.59)	3.21	0.07
Non-survivor	25(6.91)	21(11.41)		

### Laboratory parameters in derivation cohort

Laboratory examination showed that the median CK levels among non-survivors (245.74, IQR: 134.98–511.15) were significantly higher than those among survivors (92.33, IQR: 62.45–139.59) (P < 0.0001). The median CK-MB levels among non-survivors (20.83, IQR: 13.50–26.80) were significantly high than those among survivors (12.09, IQR: 9.76–16.00) (P < 0.0001). The median NT-proBNP level was 221 pg/mL (IQR: 71.75–560.75 pg/mL). NT-proBNP levels among non-survivors were significantly higher than those among survivors (P < 0.0001). Levels of Mb, LDH, PCT, WBC and LAC of non-survivors were significantly higher than those of survivors (P < 0.0001) (Table 2).

**Table 2:** Comparison of the laboratory parameters between survivors and non-survivors in derivation cohort.

Factors	Outcomes (N = 362)		*Z*	*P*
	Survivors(n = 337)	Non-survivors(n = 25)		
LDH	272.00(238.05~321.00)	347(302~531)	−4.77	<0.001
CK	92.33(62.45~139.59)	245.74(134.98~511.15)	−5.27	<0.001
CK-MB	12.09(9.76~16.00)	20.83(13.50~26.80)	−4.42	<0.001
Mb	25.58(12.15~53.45)	133.37(46.56~497.27)	−5.32	<0.001
PCT	0.07(0.05~0.18)	0.48(0.16~1.61)	−5.07	<0.001
GLU	5.80(5.10~6.85)	10.70(6.85~17.00)	−5.55	<0.001
WBC	9.76(7.69~12.96)	17.34(13.87~20.73)	−5.97	<0.001
LAC	1.30(0.90~1.80)	4.60(2.40~9.10)	−5.62	<0.001
NT-proBNP	198.00(63.00~465.00)	5302.00(1523.00~17482.00)	−6.92	<0.001

LDH: Lactate Dehydrogenase; CK: Creatine Kinase; CK-MB: Creatine Kinase-MB; Mb: Myohemoglobin; PCT: Procalcitonin; GLU: Glucose; WBC: White Blood Cell; LAC: Lactate; NT-proBNP: N-terminal pro brain natriuretic peptide.

Continuous variables were converted into categorical variables to establish the mortality risk score for severe HFMD by logistic regression. As shown in the univariate analysis, laboratory parameters associated with non-survivors of severe HFMD included LDH (OR = 8.34, 95%CI: 3.18–21.90), CK (OR = 7.73, 95%CI: 3.27–18.30), CK-MB (OR = 5.00, 95%CI: 1.99–12.57), Mb (OR = 10.97, 95%CI: 4.58–26.26), PCT (OR = 6.15, 95%CI: 2.64–14.33), Glu (OR = 18.56, 95%CI: 7.31–47.15), WBC (OR = 17.22, 95%CI: 7.06–42.01), LAC (OR = 42.63, 95%CI: 16.02–113.47), 1300 pg/mL ≤ NT-proBNP ≤ 10000 pg/mL (OR = 49.42, 95%CI: 14.55–167.85), and NT-proBNP > 10000 pg/mL (OR = 140.85, 95%CI: 32.31–614.02) (Tables 3 and 4).

**Table 3:** Univariate analysis of laboratory parameters for severe HFMD patients in derivation cohort.

Laboratory parameters	Range	Outcomes (N = 362)		*χ* ^2^	*P*
		Survivors(n = 337)	Non-survivors(n = 25)		
CK(U/L)	≤174	274 (96.82%)	9 (3.18%)	28.00	<0.001
	>174	63 (79.75%)	16 (20.25%)		
CK-MB(IU/L)	≤24	308 (94.77%)	17 (5.23%)	13.88	<0.001
	>24	29 (78.38%)	8 (21.62%)		
LDH(IU/L)	≤450	319 (94.94%)	17 (5.06%)	24.81	<0.001
	>450	18 (69.23%)	8 (30.77%)		
Mb(ng/L)	≤90	290 (96.99%)	9 (3.01%)	40.56	<0.001
	>90	47 (74.60%)	16 (25.40%)		
PCT(ng/ml)	≤0.5	293 (95.75%)	13 (4.25%)	21.73	<0.001
	>0.5	44 (78.57%)	12 (21.43%)		
NT-proBNP(pg/ml)	≤1300	313 (98.74%)	4 (1.26%)	136.17	<0.001
	1300~10000	19 (61.29%)	12 (38.71%)		
	>10000	5 (35.71%)	9 (64.29%)		
Glu(mmol/L)	≤7.8	296 (97.69%)	7 (2.31%)	61.07	<0.001
	>7.8	41 (69.49%)	18 (30.51%)		
WBC(10^9^/L)	≤16.5	310 (96.88%)	10 (3.13%)	61.33	<0.001
	>16.5	27 (64.29%)	15 (35.71%)		
LAC(mmol/L)	≤3.2	321 (97.57%)	8 (2.43%)	112.39	<0.001
	>3.2	16 (48.48%)	17 (51.52%)		

CK:Creatine Kinase; CK-MB: Creatine Kinase-MB; LDH: Lactate Dehydrogenase; Mb: Myohemoglobin; PCT: Procalcitonin; NT-proBNP: N-terminal pro brain natriuretic peptide; GLU: Glucose; WBC: White Blood Cell; LAC: Lactate.

**Table 4:** The analysis of possible and independent laboratory parameters in the derivation cohort and the mortality score of severe HFMD patients.

Factors	Univariable analysis		Multivariable analysis			Point value
	OR(95%CI)	Sig	OR(95%CI)	Sig	B	
LDH(IU/L)	8.34(3.18,21.90)	<0.001	0.80(0.06,10.63)	0.86	−0.23	
CK(U/L)	7.73(3.27,18.30)	<0.001	2.37(0.48,11.86)	0.29	0.86	
CK-MB(IU/L)	5.00(1.99,12.57)	<0.001	0.15(0.01,2.45)	0.18	−1.91	
Mb(ng/L)	10.97(4.58,26.26)	<0.001	3.04(0.55,16.97)	0.21	1.11	
PCT(ng/ml)	6.15(2.64,14.33)	<0.001	0.64(0.12,3.52)	0.61	−0.45	
Glu(mmol/L)	18.56(7.31,47.15)	<0.001	4.26(1.01,18.10)	0.04	1.45	1
WBC(10^9^/L)	17.22(7.06,42.01)	<0.001	8.57(1.96,37.53)	<0.001	2.15	2
LAC(mmol/L)	42.63(16.02,113.47)	<0.001	9.40(1.83,48.20)	0.01	2.24	2
1300 pg/ml ≤ NT-proBNP ≤ 10000 pg/ml	49.42(14.55,167.85)	<0.001	9.08(1.75,47.16)	0.01	2.21	2
NT-proBNP > 10000 pg/ml	140.85(32.31,614.02)	<0.001	37.62(4.56,310.39)	0	3.63	4

### Development of mortality risk score for severe HFMD

For the nine significant laboratory parameters (P < 0.05) found during univariate analysis, an unconditional logistic regression model was used for multivariate analysis. The backward method was used for screening the variables. In multivariate analysis, we demonstrated that NT-proBNP, GLU, WBC, and LAC were significantly associated with non-survivors of severe HFMD. Patients with GLU > 7.8 mmol/L had higher mortality risk than those with GLU ≤ 7.8 mmol/L(OR = 4.26, 95%CI: 1.01–8.10). Severe HFMD patients with WBC > 16.5 × 10^9^/L had higher mortality risk than those with WBC ≤ 16.5 × 10^9^/L (OR = 8.57, 95%CI: 1.96–37.53). Patients with LAC > 3.2 mmol/L had higher mortality risk than those with LAC ≤ 3.2 mmol/L(OR = 9.40, 95%CI: 1.83–48.20). Patients with NT-proBNP ≥ 1300 pg/mL and NT-proBNP ≤ 10000 pg/mL had higher risk of death than those with NT-proBNP < 1300 pg/mL(OR = 9.08, 95%CI: 1.75–47.16). Patients with NT-proBNP levels of >10000 pg/mL had higher mortality risk than those with NT-proBNP < 1300 pg/mL (OR = 37.62, 95%CI: 4.56–10.39). The regression model had good discrimination of non-survivors with severe HFMD, with AUC of 0.97 (95%CI: 0.94–1.00), and results of the Hosmer-Lemeshow goodness-of-fit test for logistic regression confirmed that the mortality risk score was well calibrated (χ^2^ = 3.92, P = 0.56).

**Figure 1:**
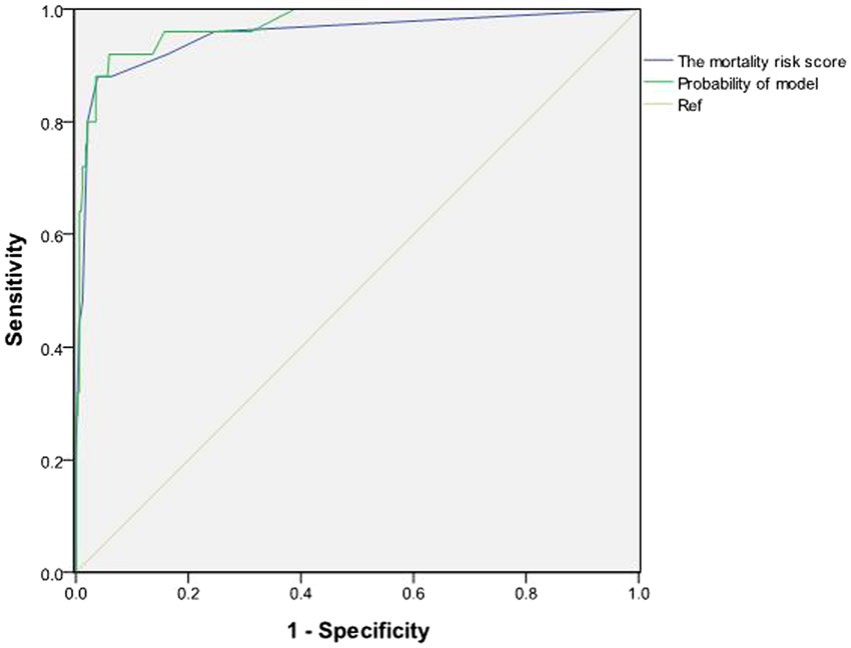
Receiver operating characteristic (ROC) curve analysis in the derivation cohort.

We then developed the mortality risk score for severe HFMD, including four laboratory parameters that independently predicted non-survivors of severe HFMD in multivariate analysis, using the regression coefficients (Table 4). A regression coefficient of 1.45 corresponded to approximately 1 point, and the resultant beta coefficients and point value for each variable were reported (Table 4). A total score was calculated by summing the points from each variable for each patient. The mortality risk score ranged from 0 to 9 points for severe HFMD patients. AUC for the mortality risk score was 0.95 (95%CI: 0.89–1.00), showing that the mortality risk score has good discrimination (Fig. 1). Compared with the AUC of 0.97 (95%CI: 0.94–1.00) determined by the logistic regression model, the AUC for the mortality risk score showed no significant difference by the C-statistic (Z = 0.55, P > 0.05).

As the mortality risk score increased, the mortality risk for severe HFMD increased (Table 5). In the present study, the maximum value of Youden’s index was considered the “optimal” cut-off point. Thus, using the “optimal” cutoff point of 4, the sensitivity, specificity, positive predictive value and negative predictive value were 88.00%, 96.14%, 62.86% and 99.08% in the derivation cohort, respectively. On the basis of similar magnitudes of risk, scores 0 through 4 were combined into a low-risk category, and scores of more than 4 were combined into a high-risk category (Table 5). In the derivation cohort, 92.54% was considered low risk, whereas 7.46% was regarded as high risk. The proportion of deaths in the high risk group was 74.07%, and that proportion in the low risk group was 1.49% (χ^2^ = 204.74, P = 0.0001).

**Table 5:** Risk for non-survivor HFMD patients by the mortality score and risk category in derivation and validation cohorts.

Risk score	Number	Death n(%)	Risk category	Total number n(%)	Death n(%)
Derivation cohort				362(100.0)	25(6.91)
0	255	1(0.39)	Low(0–4)	335(92.54)	5(1.49)
1	29	1(3.45)			
2	35	1(2.86)			
3	8	0(0.00)			
4	8	2(25.0)			
5	11	8(72.73)	High(>4)	27(7.46)	20(74.07)
6	3	1(33.33)			
7	8	6(75.00)			
8	2	2(100.00)			
9	3	3(100.00)			
Validation cohort				184(100.00)	21(11.41)
0	101	1(0.99)	Low(0–4)	166(90.22)	7(4.22)
1	12	0(0.00)			
2	37	2(2.54)			
3	7	1(14.29)			
4	9	3(33.33)			
5	4	3(75.00)	High(>4)	18(9.78)	14(77.77)
6	3	1(33.33)			
7	9	9(100.00)			
8	1	0(0.00))			
9	1	1(100.00)			

### Internal and external validation

**Figure 2:**
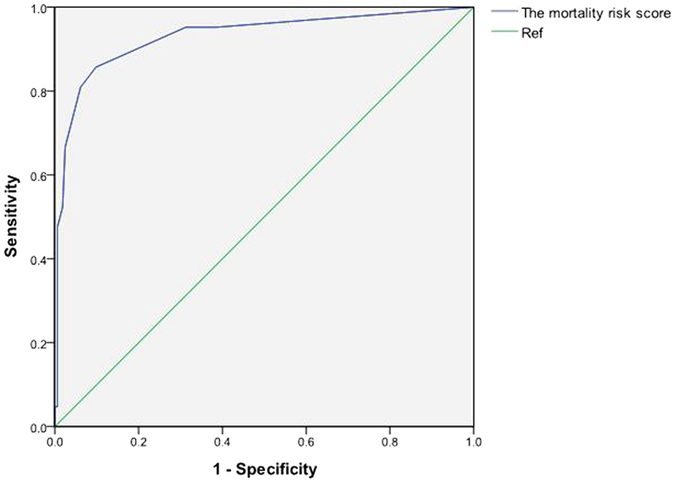
Receiver operating characteristic (ROC) curve analysis in the validation cohort.

The average AUC obtained by internal validation using the bootstrap method was 0.93 (95%CI: 0.91~1.00), which is similar to that from the derivation cohort. The average AUC by internal validation demonstrated that the mortality risk score for severe HFMD had good discrimination. In the validation cohort, the mortality risk of severe HFMD patients also increased as the points increased (Table 5). With the same mortality risk categories as defined in the derivation cohort, 166/184 (90.22%) patients were considered as low risk, whereas 18/184 (9.78%) of patients were considered high risk. The proportion of deaths among patients with severe HFMD was 4.22% and 77.77% for the low- and high-risk groups (χ^2^ = 86.39, P = 0.0001). The high risk group accounted for 80.95% of all severe HFMD patients who died. AUC for the mortality risk score was 0.93 (95%CI: 0.86–1.00) in the validation cohort, which did not differ statistically from that of the derivation cohort (Z = 0.14, P > 0.05) (Fig. 2). According to the “optimal” cutoff in the derivation cohort, the sensitivity, specificity, positive predictive value and negative predictive value were 80.95%, 93.83%, 62.96% and 97.44% in the derivation cohort, respectively. In addition, the results of the Hosmer-Lemeshow goodness-of-fit test for logistic regression confirmed that the mortality risk score was well calibrated in the validation cohort (χ^2^ = 3.92, P = 0.56).

## Discussion

Based on the national surveillance system of HFMD in China, the case severity rate of HFMD is 1.1%, and the case-fatality of rate is 0.03%^17^. In Asia, many countries have experienced increasing trends of HFMD outbreaks with deaths among younger children owing to cardiorespiratory dysfunction^18^. Thus, severe HFMD is a major public health concern in China, and a serious threat to the health of Chinese children. In this study, we developed and validated a mortality risk score for severe HFMD that can be used to provide effective medical intervention, and reduce the mortality rate among severe HFMD patients. Our major findings are as follows: (1) The mortality risk score for severe HFMD, which comprised four simple, readily available objective laboratory parameters (WBC, GLU, LAC and NT-proBNP) can provide accurate risk stratification for severe HFMD patients in intensive care unit. (2) The mortality risk score demonstrated good discrimination and calibration in our derivation ad validation cohorts by the AUC and Hosmer-Lemeshow chi-squared statistic. (3) The mortality risk score may lead to better stratification for severe HFMD patients with a high risk of death. On the basis of similar magnitudes of risk, scores 0 through 4 were combined into a low-risk category, and scores of more than 4 combined into a high-risk category.

Some risk factors identified in previous studies, such as young age, rural residence, the level of hospital at first visit, some subjective clinical manifestations (leg tremors, lethargy, papular rash, and circulatory disturbance) and human EV-71 infection were not included in the mortality risk score^17, 19^. For one thing, no “gold standard” clinical manifestations, such as lethargy or circulatory disturbance, can be identified in a timely and effectively manner. Moreover, previous studies have demonstrated that EV71 is more likely to cause serious complications than other enteroviruses and leads to lesions of the central nervous system^20^. Because EV71 is a more virulent strain of enterovirus, it is more likely to induce viremia, and complications, such as meningoencephalitis, pulmonary haemorrahage, and circulation failure^3, 21^. However, etiological examination requires special equipment and is time-consuming, making this application difficult in general medical practice or for every patient. Early recognition using a risk score based on laboratory parameters collected 1 hour after hospital admission is extremely useful. Therefore, the present mortality risk score was developed for the early recognition of patient with severe HFMD, to be able to provide effective and timely medical intervention.

In the present study, we found that severe HFMD patients with elevated WBC counts or GLU levels were more likely to die than those without leukocytosis or hyperglycaemia. This finding is similar to several previous studies, in which leukocytosis and hyperglycaemia were considered two risk factors for fatalities in EV71-associated HFMD^22, 23^. Li *et al*.^24^ study demonstrated that in mild HFMD cases where GLU levels rapidly increased, these cases would develop into severe cases within 2–4 hours^24^. Hyperglycaemia was observed in fatalities in the present study, which may have resulted from the loss of blood glucose homoeostasis. Blood glucose homoeostasis is affected by the autonomic nervous system. Upon stimulation of the sympathetic nervous system, blood adrenaline and glucagon concentrations increase, and insulin concentrations decrease^25^. The GLU levels can be mediated by the action of adrenaline or glucagon on the liver, and by direct innervations of the liver^26^. Although previous studies have demonstrated that leukocyte count and serum glucose can predict the severity of HFMD, these are late symptoms that generally occur with fulminant disease and have the problem of being non-specific and poor correlation with neurological complications^27, 28^. Thus, for timely, systematic and comprehensive assessment among HFMD patients, a mortality risk score that includes relevant laboratory parameters is required.

We demonstrated that the mortality risk of patients with severe HFMD who had blood LAC levels >3.2 mmol/L was 9.40 times greater than those with LAC levels ≤ 3.2 mmol/L. This finding is similar to that of Liu *et al*. (2015) study, in which elevation of blood lactic acid was an independent mortality risk factor among severe HFMD cases^29^. Blood lactate concentrations produced in muscle, the intestines, red blood cells, the brain, and other organs have been used widely as a marker of oxygenation and circulatory sufficiency in critically ill patients^30^. Several previous studies have demonstrated that elevated blood glucose levels and hyperlactataemia can not only reflect the severity of illness, but can also be used to predict the prognosis of critically ill patients^28, 31^. Similar to blood glucose levels, elevated blood lactic acid levels may suggest poor peripheral perfusion or more advanced disease status.

Our research demonstrated that the serum NT-proBNP level was the most important of the four independent risk factors after multiple logistic regression analysis. In China, some studies have demonstrated that NT-proBNP and B-type natriuretic peptide levels vary among HFMD patients with different stages of disease or different complications, which may reflect the changes in heart function at different stages of HFMD in children^32, 33^. Serum NT-proBNP levels can reflect the cardiovascular status of patients with early-stage severe HFMD and may help clinicians to identify patients with worse prognosis before they develop pulmonary oedema, pulmonary haemorrhage, or heart failure.

Any scoring system requires updating and ongoing prospective evaluation to enhance its generalizability and acceptance. Our results showed that the present mortality risk score for severe HFMD had good discrimination and calibration in the derivation and validation cohorts. The performance indices obtained from internal and external validation were nearly the same as those from the derivation cohort. These findings suggest that the mortality risk score is robust and valid and likely has generalizable discrimination in varied settings across China. In addition to its validity and accuracy, we have demonstrated that the mortality risk score is also credible. First, all the main known mortality risk factors of laboratory parameters were considered in the analysis, and no obvious items were missing. Second, the mortality risk score, comprising WBC counts, GLU, blood LAC levels, and NT-proBNP, were consistent with the results in previous studies^9, 13^. Finally, the mortality risk score was established using recommended methods that are widely used in the derivation and validation of a prediction rule.

In addition, the mortality risk score comprised only four readily available laboratory parameters, which may potentially facilitate recognition of high mortality risk in severe HFMD cases, and be widely applicable to clinical care and research settings. With regard to potential clinical application, the mortality risk score can serve a dual purpose in assessment. First, the score can be used to identify high-risk cases in PICU so that prophylactic measures can be taken to reduce the case fatality rate. Second, the mortality risk score may also be applied in the emergency department to improve triage decisions.

There are some limitations in our study. First, this was a small, single-centre, prospective cohort study and had inherent limitations that are common to all observational studies. Although we performed external validation, the generalizability of the mortality risk score to the general population remained questionable. The mortality risk score should be validated in a multisite external population in further studies. Second, for the exclusion of patients, the patient age, disease stage, aetiology, and length of ICU stay were not recorded. Therefore, selection bias exists in our study. Third, simple, readily available, and objective laboratory parameters were included to establish the mortality risk score for severe HFMD. Some typical and subjective clinical manifestations, like peak temperature of 38.5 °C or more, vomiting, lethargy, circulatory disturbance, and dysfunction of respiratory rhythm, were not considered as the risk factors for death in this study. Lastly, four simple, and objective laboratory parameters (WBC, GLU, LAC and NT-proBNP) were used to establish the mortality risk score for severe HFMD. However, because NT-proBNP is expensive and not widely available in primary care settings, the generalizability and application of the mortality risk score would be limited in that setting.

## Patients and Methods

### Ethics statement

This study was approved by the Ethics Committee of Hunan Children’s Hospital (IRB No. HCHLL-2014004). Informed written consent was obtained from the parents or caretakers of each child included in the study. All data collection from participants was fully anonymous. All experiments were performed in accordance with the approved guidelines and regulations.

### Patients

This study was designed as a retrospective cohort study conducted in Hunan Children’s Hospitals, which serves a population of 71 million and a land area of 211, 800 km^2^. Hunan Province is located in central China and a population of 11.574 million children was recorded in 2013.

All participants included in the present study were patients with HFMD who were admitted to the PICU between 1 January, 2012 and 31 December, 2014. The diagnostic criteria used were based on the Chinese guidelines for HFMD diagnosis and treatment issued by the Ministry of Health of China^34^. Children were diagnosed with typical HFMD if they had at least one of the following features: a maculopapular or vesicular rash on the palms, soles,or buttocks and vesicles or ulcers in the mouth. Atypical disease was defined as exanthema on distinct sites other than the oral/perioral region, palms of the hands and soles of the feet. Typical or atypical HFMD patients were diagnosed based on initial experience and virological detection of EV71 and CA16 by two experts who had worked in the PICU for 5 years. Cases of severe HFMD were diagnosed if patients were considered to have more serious complications including encephalitis, meningitis, acute flaccid paralysis (AFP), and cardiorespiratory failure. All severe HFMD patients had been admitted to the PICU, and we excluded patients if their clinical presentation could be explained by another specific illness, such as varicella, herpes simplex virus, herpes zoster, contact dermatitis, atopic dermatitis, bullous impetigo, or other bullous diseases. Twenty-six HFMD patients with incomplete medical records who were transferred to other tertiary hospitals or whose parents withdrew their child were also excluded; 10 patients were from derivation cohort, and 16 patients were from validation cohort. Finally, we enrolled 546 patients as participants in the present study. Each participant received a chart recording age, sex, diagnosis, lactate dehydrogenase (LDH), creatine kinase (CK), creatine kinase-MB (CK-MB), myohemoglobin (Mb), procalcitonin (PCT), lactate (LAC), white blood cell (WBC) count, and glucose (GLU). All physiological parameter values were collected during the first hour after admission to the PICU in this study. Levels of amino-terminal fragment of the prohorrmone brain-type natriuretic peptide (NT-proBNP) were determined using an electro-chemo-luminescence immunoassay (Elecsys NT-proBNP, bio Mérieux, Lyons, France).

### Derivation and validation cohorts

To develop the mortality risk score, all patients with severe HFMD who were admitted to PICU between 1 Jan 2012 and 31 Dec 2013 were recruited as the derivation cohort. To validate the risk score, all patients with severe HFMD admitted to PICU between 1 Jan 2014 and 31 Dec 2014 were recruited as the validation cohort. The validation cohort was not used until after the multiple logistic regressions model and scoring system had been created.

### Definitions

The definition of severe HFMD was based on the Guidelines for HFMD Diagnosis and Treatment, version 2010, published by the National and Family Planning Commission^34^. Patients were diagnosed with severe HFMD if they were considered to have more serious complications including encephalitis, meningitis, acute flaccid paralysis (AFP), cardiorespiratory failure, or died. Meningitis was defined as pleocytosis in the cerebrospinal fluid. All HFMD patients were divided into four subgroups based on the clinical stage of their disease during the course of hospitalisation^35^. Encephalitis is characterized by impaired consciousness, overt afebrile seizure, or focal neurological deficit. Specifically, brainstem encephalitis is characterized by frequent myoclonic jerks or AFP. AFP is characterized by the acute onset of areflexic limb weakness. Cardiorespiratory failure is defined as the presence of respiratory distress, tachycardia, pulmonary oedema, and pulmonary haemorrhage. Pulmonary oedema is defined as patients who have respiratory distress and is confirmed by chest X-ray findings. Pulmonary haemorrhage is defined as chest radiographs showing alveolar congestion plus pink frothy fluid or blood from the endotracheal tube.

### Statistical analysis

Data was collected using Epidata 3.2 (EpiData Association, Odense Denmark) and analysed using Statistical Package for the Social Sciences, Windows version 18.0 (SPSS Inc., Chicago, IL, USA). Continuous variables were described using means and standard deviations if the data were normally distributed. A Student *t* test was used to compare the differences in continuous variables. Continuous variables were described using median and inter-quartile range (IQR) if the data were not normally distributed. The Mann-Whitney U test was used to compare the differences in physiological parameters between the survivor and non-survivor groups. Continuous variables were converted into categorical variables. All categorical explanatory variables were examined by univariate analysis using a chi-squared (χ^2^) or Fisher’s exact test. Variables with *P* < 0.05 in the univariate analysis were selected as candidates for the multivariate logistic regression analysis. Using backward stepwise selection (likelihood ratio test), a multivariate logistic regression analysis was performed to develop a preliminary model. To predict the mortality risk of severe HFMD in a given individual, we designed a prediction rule with the independent variables selected by multivariate analysis. A simple integer-based point score for each predictor variable was established by dividing beta coefficients by the absolute value of the smallest coefficient in the model and rounding up to the nearest integer. The total score for each participant was calculated by adding each component together. The receiver operating chartacteristic (ROC) curve and 95% confidence interval (CI) were computed. We chose the score that discriminated between a low-risk category and high-risk category as the cut-off value and then calculated the sensitivity and specificity of the mortality risk score for severe HFMD. Discrimination and calibration of the model were assessed by area under the receiver operating characteristic curve (AUC) and the Hosmer-Lemeshow chi-squared statistic in the derivation and validation cohorts, respectively^36, 37^. Discrimination between death and survival was assessed by calculating the area under the ROC curve (AUC)^37, 38^. The predictive value was classified as low (AUC = 0.5–0.7), moderate (AUC = 0.70–0.90), or high (AUC = 0.90–1.0)^39^. Calibration across deciles of risk was evaluated using the Hosmer-Lemeshow goodness-of-fit test^40^. For this test, a P-value > 0.05 indicates good calibration. We validated the prediction score internally using the bootstrap method in the original data set by sampling with replacement for 1000 iterations^41, 42^. The prediction rule was also externally validated in the independent validation cohort. Scores were calculated among all validated data as well as by using the same method. Finally, the sensitivity, specificity, and AUC were compared between derivation and validation data. The overall discriminative ability of the mortality risk score for severe HFMD was measured with the C-statistic in both the derivation and validation cohorts.
